# Reimagining brief interventions for alcohol: towards a paradigm fit for the twenty first century? INEBRIA Nick Heather Lecture 2019: This lecture celebrates the work of Nick Heather in leading thinking in respect of both brief interventions and wider alcohol sciences

**DOI:** 10.1186/s13722-021-00250-w

**Published:** 2021-06-29

**Authors:** Jim McCambridge

**Affiliations:** Department of Health Sciences, Seebohm Rowntree Building, University of York, Heslington, York, YO10 5DD UK

**Keywords:** Alcohol, Brief interventions, Primary care, Screening, Public health, Alcohol marketing, Alcohol policy, Alcohol industry

## Abstract

**Background:**

There is no longer support for the idea that brief intervention programmes alone can contribute meaningfully to the improvement of population health relating to alcohol. As a result, calls for major innovations and paradigm shifts grow, notably among research leaders.

This paper briefly examines the history of the development of the evidence-base from the landmark World Health Organisation projects on Screening and Brief Intervention (SBI) in the 1980s onwards. Particular attention is given to weaknesses in the theorisation of social influence and interventions design, and declining effect sizes over time. Although the old SBI paradigm may be exhausted where it has been applied, it has not been replaced by a new paradigm. Alcohol marketing encourages heavy drinking and today may have more powerful effects on thinking about alcohol, and about alcohol problems, than previously. The nature of the societal challenge being faced in an alcogenic environment in which alcohol is widely promoted and weakly regulated underpins consideration of the possibilities for contemporary evidence-informed public health responses. Evidence-informed perspectives in discourses on alcohol problems need to be strengthened in redeveloping rationales for brief interventions. This process needs to move away from sole reliance on a model based on a two-person discussion of alcohol, which is divorced from wider concerns the person may have. Reimagining the nature of brief interventions involves incorporating digital content, emphasising meso-level social processes based on material that people want to share, and seeking synergies with macro-level population and media issues, including alcohol policy measures.

**Conclusions:**

Current versions of brief interventions may be simply too weak to contend with the pressures of an alcogenic environment. A new generation of brief interventions could have a key role to play in developing multi-level responses to the problems caused by alcohol.

## Background

Brief interventions for alcohol have aimed to make widely available advice and counselling to heavy drinkers so that they can minimise risks to future health, avoid or manage problems in controlling consumption, and refer to treatment services if needed [1–3]. They have been developed and studied for approximately 60 years [4]. Alcohol treatment interventions have gotten briefer over time and become similar in content to brief interventions in the form of the counselling offered opportunistically to drinkers not asking for help [5–7]. Early promise led to applications for drug use and other health issues [8, 9]. With the growth of the internet, digital interventions have been developed and conceptualised in ways initially informed by similar principles to face-to-face contacts [9]. Brief interventions are not an intervention per se but a broad family, and a pragmatic way of thinking about seizing opportunities for improving public health [3, 10, 11].

## Main text

### 40 years of screening and brief intervention

A major World Health Organisation initiative which originated around 1980 established a new alcohol prevention paradigm for primary care [1]. This Screening and Brief Intervention (SBI) approach has been important, placing alcohol issues on policy agendas worldwide. It originally developed the screening tool [12], advanced a basic model for brief advice, and was non-specific about counselling [13, 14]. The former was designed for delivery in as little as 5 min and the latter afforded 20 min in the SBI paradigm, which was then extended to other health and non-health settings [11, 15].

The conceptual basis of advice was stated as follows: “Simple advice was chosen as the minimal intervention to determine whether social influence, as communicated through firm advice to modify unhealthy drinking, would be sufficient to motivate patients to modify their drinking”[14]. It is unlikely that the articulated conception of the transmission of social influence would have withstood scrutiny from social scientists at the time. Similarly: “The brief counseling strategy was chosen to evaluate whether drinking may be even more amenable to change when behavioral techniques are added to social influence” [14].

Conceptual limitations of the SBI paradigm were recognised early, particularly for problem recognition [16]. Behavioural techniques were subsequently rarely applied in brief intervention innovations [17], partly as the approach of Motivational Interviewing [18, 19] came quickly to dominate thinking about alcohol counselling. This was likely because there were clear but not well understood challenges in talking about drinking in a constructive atmosphere. There have been few innovations in advice, which continues to be conceptually crude and has not been meaningfully informed by process study [20]. Recommended forms of advice are unappealing to key practitioner groups as they pay little or no attention to tailoring to individual concerns [7, 21].

There are many puzzling findings in the SBI literature, amongst which is the lack of superiority of apparently more sophisticated interventions over cruder ones [22], perhaps because they are more challenging to learn and deliver well. Efforts to address longstanding weaknesses of the literature have not made major advances. For example, almost 30 years ago, Bien and colleagues [6] highlighted the need to better understand mechanisms and mediators, how they work, and for whom, particularly in terms of the level of drinking or extent of problems that might reasonably be expected to be amenable to such intervention. There remains major uncertainties about all these matters today, including the latter key issue [23]. Some progress has been made in understanding mechanisms of effects in motivational interventions [24].

Over time effect sizes have reduced, with the most recent Cochrane primary care review indicating a 20 g per week reduction in drinking after 12 months, compared to 38 g in the first version; on average declining 2.3 g per week per trial publication year[22]. Accordingly, doubts have grown about the benefits, if any, that can be realistically anticipated in routine practice [21]. Effect size reduction appears partly due to attenuation of publication bias more recently [22], though overall differences continue to be influenced by large effects in less strongly designed studies in settings other than general practice.

It has been proposed that greater scrutiny of the limitations of existing evidence may provide a basis for recalibrating expectations and constructing new research agendas [21]. Reliance on self-reported outcomes is one key limitation that has invited scepticism. For example, close attention to effect sizes in the small number of studies with GGT outcomes in the Cochrane primary care review [22] shows them to be smaller than for self-reported outcomes in the same studies. It must be acknowledged that the issues rather briefly discussed here are important and complex, and not widely studied, so readers are encouraged to engage critically with the observations made here. See Box 1 for some study possibilities, some of which are quite typical of meta research, identifying influences on the evolution of literatures that are not specific to brief interventions, and others that are more or less specific. In this essay, I address the prospect that early theoretical weaknesses are particularly problematic for brief interventions in a more challenging period.

Until recently, research translation has been weak in most countries, in part because of evidence limitations, with briefer interventions than have been studied being implemented in practice due to time pressures in busy health systems [25]. Studies investigating the training of practitioners to deliver brief interventions have been much less prominent than evaluations of SBI per se, and such studies reveal the major challenges involved [26]. This is difficult work to do well and routine training provision has been little studied, though it is not difficult to find examples which depart so considerably from the available evidence that any benefit seems unlikely. The causal chain from training practitioners to impacting drinking behaviour is long [27], and the evidence on how to do this limited.

The fact that training provisions exist reflects the key agenda-setting function that has been successfully fulfilled by SBI, informing thinking about how health systems may secure health maintenance in the populations they serve. As rising burdens of alcohol and other pressures on health systems have grown, SBI has offered an attractive and charismatic, apparently strategic, vehicle for implementation. There is a painful irony that large national programmes have been developed and implemented in many countries only in recent years, in an era when many in the research community increasingly regard the 1980s SBI paradigm as now having been exhausted.

There is no longer support for the idea that brief intervention programmes alone can contribute meaningfully to the improvement of population health; this requires alcohol policies [15]. Calls for major innovations and paradigm shifts grow, notably among key research leaders [28, 29]. Such perspectives emphasise the unmet needs of drinkers experiencing problems controlling consumption, connections to existing health problems, and consequently greater appreciation of the complexities inherent in the burdens of alcohol on health systems [21]. According to Andreasson:

“Practitioners have always been reluctant to do screening. We need to find smarter ways to initiate discussions about alcohol use; more related to clinical relevance and patient concerns, recognizing that alcohol use contributes to much of the morbidity encountered in primary care…the right response is not to throw out the baby with the bathwater” [30].

It now seems obvious that addressing alcohol consumption in isolation from appreciation of the person’s circumstances is unwise, at least when one has the opportunity for discussion.

Over the last decade or so, internet and other digital interventions show great promise, with effects larger than now seen with face-to-face interventions [31, 32]. This is in some ways counter-intuitive. It is possible that this approach is beneficial because it avoids some of the pitfalls in talking about drinking (see below), yet more limited capacity to explore a person’s situation suggests that caution about the evidence is needed. Appreciation of risk of bias rarely includes attention to the validity of self-report being differential between randomised arms, though there are exceptions e.g. [31]. In online trials the largest studies mainly produce null results [32], and larger trials are usually more reliable than smaller studies. It will be interesting to see how far this newer literature follows a similar trajectory to the older face-to-face one, with effect sizes diminishing over time.

### Taking stock of social influence

The theoretical weakness of brief interventions has profound effects. Understanding of the scale and nature of the population health and societal problems caused by alcohol has grown enormously over the past 40 years [33]. Public health and social scientists would note brief interventions pay little attention to the social contexts of individual decision-making, and are largely implemented in isolation from population-level interventions. The alcohol policy measures that evidence shows would help populations to reduce alcohol problems, in part by operating through individual decision-making, have been hard to implement globally [34].

Neoliberal ideas that seek to reduce the role of the state, and promote consumption of commodities delivered by the market, ostensibly enhancing personal freedom, dominate instead. These entail the widespread availability of alcohol, and the unregulated promotion and normalisation of heavy drinking [35]. Corporate influences operating through alcohol marketing mould individual decision-making on drinking, so that one is encouraged to express individuality as a consumer. At the same time one is encouraged to take responsibility for one’s health, whilst paradoxically actually deciding to drink less is harder to implement in practice, with pro-consumption social influences pushing in the opposite direction [35, 36]. These contradictory pressures on the individual culminate in expectations about drinking, and indeed about behaviour and health more widely. According to Reith:

“The focus on the (flawed) individual consumer downplays the role of big business in producing excess, and the role of governments and regulators in creating the political conditions for them to do so…The ideology of responsibility rests on judgements about autonomy and rationality, and in it, responsible consumption is evidence of the ‘right’ way to live…Indeed, it becomes the duty of sovereign consumers to furnish themselves with relevant knowledge and information and take appropriate steps to safeguard their health” [36]

Conceptual work on brief interventions needs to encompass attention to how social influences can be understood, as they inescapably intrude upon conversations about alcohol. Indeed they shape how people think about alcohol, the associated risks and harms, as well as about the personal and societal responses needed. Comments such as “I know what I should do, but…” are not infrequently encountered in discussions with people about their drinking, perhaps more so the heavier it is. The conversations had in the course of brief interventions delivery are loaded with these kinds of influences. They exert pressures that apply to all forms of consumption that have deleterious health consequences, for example obesity is framed as an individual problem in similar ways [37]. It has been suggested that they may apply with special force to alcohol, and other addictive forms of consumption, in part due to the legacy of older ideas on addiction and personal responsibility [35].

The moralization of individual drinking provides a key role in arguments by transnational corporations that play down the scale of alcohol problems and blame a minority for them, and thereby oppose alcohol policy measures [34]. Success in so doing in turn reinforces corporate power to produce, market and sell more alcohol, making autonomous decision-making more difficult still. The more heavy drinking and alcohol problems become concentrated in populations with other kinds of problems as respite, the easier it is to blame, pathologise and coerce this minority. This reinforces the seeming logic of targeting within policy making, notwithstanding ineffective outcomes, and permits unfettered market access to the majority. This vicious circle can be thought of as a kind of neoliberal trap, in which the apparent freedoms for both individuals and populations are illusory.

The neoliberal trap may be a twenty-first century version of the embrace of disease thinking, which located problem drinking entirely within the individual in the twentieth century. Then, as now, industry actors were eager to ally themselves to efforts to respond to problems [38], thereby rendering invisible their own responsibility for problems. Attention to the sub-group of people with the X-factor who could not control their drinking was then and now juxtaposed to the interests of the rest of society who could be relied upon to drink normally [35]. Such a perspective is fundamentally in competition with the population health perspective that alcohol is a potentially dangerous drug that can be expected to cause extreme problems in a small minority of the population, and less severe problems among a much larger segment of the population [39]. The original idea of brief intervention as a population health intervention emphasised the accumulation of numbers of individuals at risk and potentially benefiting [1, 3], more so than synergies with population-level interventions. Brief interventions thus need to contend with both the hidden morality that is part of the backdrop to consideration of one’s own drinking, and stereotypical ideas that impede thinking and talking about the influences on one’s own behaviour. Twenty-first century social influences thus may well make the work of brief interventions harder to do, and this may be part of the explanation for reducing effect sizes.

### Considerations informing public health responses

Thinking about brief interventions needs to better locate itself within contemporary public health, which appreciates social determinants of health. The shape of brief, and indeed other individual-level, interventions, within multi-level responses motivated by public health considerations, in competition with alcohol marketing and other social influences acting to promote consumption, remains to be clarified. What follows is a brief sketch of some preliminary thinking.

There is a need, firstly, to recognise the scale, and better understand the nature, of the challenge. Addictive consumption is the most profitable form of consumption, and the externalities are not borne by the corporation unless concerted actions are taken by policy makers, which may be rare. Such issues form part of emerging agendas on the commercial determinants of health [40]. The prevalence of alcohol consumption and other addictive behaviours is on the rise globally as weakly regulated corporate forces specifically seek to increase consumption in new populations, aided by ever more sophisticated digital possibilities for promotion [36]. Persuasion industries have grown quickly, using new technologies to offer hitherto remarkable means of accessing and influencing individual thinking and behaviour.

There is a need also to accept this challenge if the adverse consequences for public health are to be arrested. This may be done by contesting the responsibility narrative that blames the individual for drinking too much, by drawing attention to the social determinants operating on the individual. This also requires, however, innovations in conceptual frameworks, specifically in order to locate corporate forces inimical to health, as they are almost entirely absent [41], as well as to identify how they play out in respect of the specific circumstances of alcohol [42]. There is a related need for assertive policy making in the interest of public health [43], based on more advanced understanding of alcohol public health decision-making processes, and how corporations seek to influence them. Multi-level theories are needed.

The public is the key constituent in thinking about public health. There is a major ideational contest over the changing, and in many ways more challenging, nature of alcohol problems in the minds of the public and policy makers. So where do brief interventions fit in? Perhaps the shortest answer is nowhere straightforwardly. Regardless of whether stronger public health policy making is attainable, however, brief intervention programmes that help individuals contend with the pressures to drink more will be needed, perhaps more so over time. This means careful study of the extent to which they are effective is needed. Indeed it may be the case that growing understanding of the benefits and limitations of brief interventions may inform considerations of approaches capable of having appreciable population-level effects. These seem likely to involve brief intervention programmes operating in synergy with other population-level interventions, in addition to the different rationale for offering help to people who need it [15]. We have a large literature available, and attention paid to conflicting findings may be particularly well rewarded.

### Thinking about alcohol problems in reworking the logic of brief interventions

Structural forces set cultural parameters around individual decision-making, and limit agency and the potential of interventions operating only at that level. As alcohol consumption remains a choice made by individuals, one may hypothetically choose to intervene at that level, or not. Such decision-making likely implies trade-offs among a range of interventions that public health planners may consider. Pressures on the financing of health systems mean that service provision is increasingly restricted for the large numbers of people who struggle with alcohol, with much suffering to themselves and others resulting. Highly stigmatised services offering help in the form of brief treatments are the norm globally [44], where they exist at all. Treatment demand is largely driven by desperation, complex mixes of alcohol and other psychosocial problems, and formal and informal compulsion [45]. Service provision for harmful drinkers (i.e. people struggling to minimise the adverse impacts of drinking on their lives) is little developed anywhere [44]. As well as responding to problems where they have become manifest, we need to find new ways of providing resources to people to help them to think about their relationship with alcohol, including whether risks are acceptable and other ways alcohol can interfere with their lives, across all levels of alcohol consumption.

The power of stereotypical ideas and the low reach of evidence-informed ideas about the nature of alcohol problems among the general population is both striking and debilitating [46]. For stigma-related reasons, the culture bound nature of ideas about loss of control, and to transcend the limitations of individualistic perspectives, it has been proposed that sustained heavy drinking over time be a focal concern of public health, particularly for defining and measuring problems [47]. This may unwittingly entail, however, a retreat from helping people to think about and to talk about the nature of the alcohol problems they experience, unless a more compelling alternative basis for understanding of the nature of alcohol problems is developed in the general public.

Widespread experiences of alcohol problems, from the relatively trivial to those that are of a more serious nature are challenging to discuss because of deeply entrenched ideas about alcoholics, alcoholism, addiction and alcohol problems of a severe kind. These ideas separate people in an unhelpful dichotomy, rendering invisible impairments that are less severe. Our culture does not support honest conversations about our own drinking, particularly so the more one drinks, because we lack the basic vocabulary, in addition to the moralization of the subject. Perhaps if we were better at talking about the small ways in which drinking interferes with our lives, we would be better placed to talk about the bigger issues? There are no easy answers to how this might be done, though recognizing it as an important task provides a first step forward.

Stereotypical ideas can reasonably be expected to continue to persist, and to provide false reassurance to heavy drinkers that their drinking is OK because it does not resemble the stereotype. Getting beyond binary thinking and developing ideas about a continuum of severity of problems, as DSM-V has endeavoured to do, should help reduce the othering of those with severe problems. Normalising discussion of the experience of alcohol problems, including those that may seem somewhat trivial such as hangovers, needs to avoid exaggerating prevalence to ward off moral panics. If so, there could be lessons to be learned from the ways in which mental health discourses increasingly recognize the widespread nature of anxiety and depression problems varying in severity, making them easier to talk about.

We are currently working on ways of opening up conversations about alcohol in the largely neglected population (from an alcohol perspective) of older adults with chronic conditions for which they are being prescribed medications [48]. This involves working with pharmacists as intervention providers and seeking to better understand their settings in order to make intervention congruent for all involved [49]. This work underscores that it is hugely challenging to strip away the moralization of the subject, for both practitioners and patients, in order to get to the beginnings of a serious consideration of whether alcohol may interfere with medication use and chronic conditions. Studies of the lived experiences of drinking and its consequences in different populations may generate new concepts of problems, and syntheses of existing data may be particularly useful. Developing understanding of how people talk about their own drinking and what they think about risk and problems may be similarly valuable [50]. Indeed, it is hard to imagine that major innovations in conversations about alcohol without it, and researchers from a range of disciplines may have contributions to make.

### Reimagining brief interventions

Individual decision-making is influenced by many factors acting proximally and distally, including those operating at different levels, so what are the implications for intervention design? It has been argued here that the conceptual horizons of brief interventions have been narrow. The effects of an alcogenic culture, with permissive norms around problems and inadequate controls on alcohol marketing are to be considered. Note also that in many countries heavy drinking traditions have deep historical roots, and these have been almost entirely ignored in intervention development. Individuals have their own richly personal experiences and views on alcohol and broader issues, as well as connectedness to those of others. Prior involvements with drinking through the lifecourse mean there are thinking and decision making resources that may be called upon in reflecting upon drinking. Helping people to think about their own drinking, or better take control of their health, in practice often involves attention to other life domains, including family, work, environment, consumption and leisure. This kind of content has not been part of, or at least not been prominent in, the brief interventions literature.

Alcohol may be important to identity formation and representation, most obviously for example among young people on social media [51]. Here alcohol marketing activity is widespread but not omnipresent, it is embedded in social networks, making it challenging to dislodge. It also potentially offers intervention material. There is scope for dissonance between the effects of unregulated marketing and glimpses of the scale and reach of the problems alcohol poses to society. For example:

“I walked through a supermarket recently and saw candles saying “Wine not?”, greeting cards with “On your marks, get set, prosecco!”, and t-shirts emblazoned with “You’ve got to begin it to win it.” When I reached the pharmacy, I saw a sign saying that alcohol is the leading cause of ill health, disability, and death among people aged between 15–49 years in the UK. It strikes me that, when it comes to alcohol, we’re living a direct recreation of the push–pull, contradictory attitudes to smoking in 1980s Britain. We knew by then that smoking was collectively killing us, yet candy cigarettes were for sale in the shops, smoking was still regarded as cool and relaxing, and those who quit smoking were sneered at for being boring, smug, and sanctimonious.” [52]

Existing evidence suggests people are much more concerned with the ways in which alcohol may cause interference in their daily lives in the here and now than with any long term health risks [53]. That might be a good place to start in assisting thinking about the nature of problems. Starting where people are, and seeing where they want to go is appropriate if one’s goal is more autonomous decision-making, less pressured by social forces such as alcohol companies. The aspiration to help people decide what is right for themselves carries profound implications for thinking about intervention; not as a process directed by an external agent, but rather as an option chosen by the person concerned. Brief interventions could thus offer resources to think about, as well as opportunities to talk about, alcohol and related issues. And not only or directly about one’s own drinking.

This perspective calls for new thinking about who to “target”, how, and with what. Rather than doing much, or perhaps any, targeting of specific content, the development of libraries of content curated so as to be accessible as resources, that people may choose to use themselves, offers a new horizon for intervention development. To do this, content has to be interesting and engaging, if the content is actually to be used. And if one is thinking about digital resources, importantly, to share with others. Social media content may help in moving from the individual-level to the meso-level to the macro-level. This implies getting quickly beyond thinking about interventions as usually involving a two person interaction.

That is not at all to imply that there is no place for discussions in health services, in the manner that brief interventions have been developed for decades. Far from it. Such discussions may, however, need to develop a much wider repertoire of brief intervention content than is already in existence, find new ways of offering it, relying more strongly on the application of broader person-centred care principles. This very likely means we need to talk about what people want to talk about. Addressing alcohol as a standalone issue, particularly when the attention is not instigated by the patient, easily falls into the moralising trap, especially when there are few problems or concerns. When alcohol may be relevant to a health concern, such as whether it interferes with medication safety or effectiveness, there is a basis for exploration of the possible connections. The rationale for any discussion need to be carefully presented in an invitation, and the decision not to take it up, respected. If an offer is accepted, the discussion then needs to be handled carefully, because of the many poorly understood influences affecting the conduct of conversations about alcohol. Fine grained research attention is needed to develop guidance for practitioners. A developing new discourse on the nature of problems may provide a vocabulary that we don’t currently possess for both practitioners and patients. This could help both parties to better understand how alcohol interferes with health or other deeply held values.

The proposal being made does, however, involve more than refining the content of existing interventions within health services. It also does more than extend this line of thinking to online interventions. Perhaps most fundamentally in proposing that brief interventions vary in the extent of personal focus. This means no longer being restricted to directly supporting the self-regulation of one’s own drinking, but also providing material that stimulates thinking about alcohol more broadly. This may have more or less direct implications for self-management, and/or sharing with others, and/or how ideas about alcohol and alcohol problems are regarded. All of which are possibilities which reconfigure the basic concept and aspirations of brief interventions, with implications for intervention aims and design. Some links providing examples of novel content from Britain and Ireland are presented in Box 2. Note such content does not rely on, and may not often involve, public health information sources.

These suggestions provide topics for conversations as well as links for sharing. Individual healthcare practitioners could invite discussion on topics they themselves have read about, or thought about, and found interesting. They might be more humble in style in not feeling obliged to seek behaviour change in delivering brief interventions. Their goals may be more realistic as a consequence. They may also be more in tune with the patient, and so be more strategic, by listening for the alcohol-related subjects that interest the patient. They can find this out by asking, though this requires vigilance for, and skill in handling, stereotypical filters to discussions, and requires comfort in having a flexible repertoire of material. Ideally, they might be able to draw on continuously updating libraries of content drawn from various sources. This prospect, however, may seem remote both as it asks a lot of busy practitioners, and such resources have not yet been developed. A more modest aspiration, and a first step, might be to develop contextually grounded patter that has the capacity to explore common reasons for attendance and alcohol, in asking how practitioners can be helpful.

## Conclusions

There are many possible futures for brief interventions, and the purpose of this piece is to extend the discussion that has already begun about the possibilities. The research agenda sketched out here is ambitious; see concrete proposals in Box 3 for taking it forward. Other views will enrich the discussion. Public health interventions are in competition with the fast evolving technologies of persuasion used in increasingly pervasive alcohol marketing, which they must endeavour to match in sophistication and capacity to influence choices being made, whilst having a fraction of the resource available. They thus may need to be much more imaginatively constructed, ambitiously proportionate to the harms caused by alcohol and pragmatically delivered, if they are to be at all impactful. They also need to support explicitly the case for stronger regulation of this drug’s production, supply and marketing [39], whilst enhancing individual capacity for more autonomous decision-making. If scientists and advocates develop new ways of talking about alcohol problems in engaging with the public, to the extent that public understanding and public opinion on policy-related issues changes, this may make alcohol policy change more likely.

To do all this, we need to eschew moralism and paternalism, and develop new narratives in key areas where they are weak, such as on the nature of alcohol problems and hidden roles of the alcohol industry in creating problems for people. Designed in the ways proposed here, a new generation of individual-level brief interventions could have stronger capacity to be integrated with population-level interventions. If public health were to abandon individual-level interventions as a fool’s errand, this means just leaving the terrain of individual decision-making to the alcohol industry, who will be keen to fill the gap. Brief interventions have always been recognised as a heterogeneous family, serving what now seems with the benefit of hindsight, rather vaguely elaborated public health purposes. Grounding thinking about the possibilities for the future of brief interventions in developments in public health sciences, and explicitly incorporating stronger engagement with social sciences, provides the basis for this proposal that the family should get bigger, and indeed become more familiar with the extended public health family.

Box 1: Why are brief intervention effect sizes declining over time? Some candidate explanations that could be empirically studied

1. Early enthusiasts and regression to the mean?
2. Stronger methods in more recent larger trials?
3. Gradual replacement of efficacy with effectiveness studies?
4. Intervention theoretical weaknesses?
5. Spin, over interpretation and promotion?
6. Cohort effects, successes of prior intervention exposures?
7. Cohort effects, failures of prior intervention exposures, target hardening?
8. Period effects, sophistication of marketing and more alcogenic environments?
9. Period effects, growing concentration of hazardous and harmful drinking in communities affected by wider health and socioeconomic inequalities?
10. Period effects, weakening of alcohol policies?

Box 2: Indicative material for innovations in brief intervention content **A thread on women, men and violence, including alcohol as a date rape drug**

- http://theconversation.com/how-alcohol-companies-are-using-international-womens-day-to-sell-more-drinks-to-women-113081
- https://www.independent.co.uk/news/uk/politics/lobbying-company-tried-to-wipe-out-wife-beater-beer-references-6284622.html
- https://en.wikipedia.org/wiki/Stella_Artois
- https://www.theguardian.com/commentisfree/2019/may/05/prince-charming-nowhere-to-be-found-in-our-toxic-sexual-lansdscape
- https://en.wikipedia.org/wiki/Reynhard_Sinaga
- https://en.wikipedia.org/wiki/John_Worboys

Box 3: Some possible next steps for researchers

1. Start studying the evolution of the brief intervention field in meta research
2. Understand the problem better; study historical trends in effect sizes in greater depth
3. Better appreciate the causal chain to beneficial outcomes by explicitly theorising brief interventions
4. Develop and explore innovations in intervention content beyond the existing paradigm; avoid restricting content to the self-regulation of one’s own behaviour alone
5. Be ambitious in developing libraries of digital content to counter marketing
6. Adopt quantitative measures of broader population needs as well as research, marketing, service provision and policy contexts
7. Explore alternatives to exclusive reliance on self-reported outcomes
8. Nest substantial qualitative studies within trials to better study contexts, implementation and outcomes
9. Use qualitative approaches outside trials to explore how people talk, and the ideas they hold, about alcohol and alcohol interventions
10. Investigate the power of longstanding stereotypical ideas about the nature of problems and how they are mobilised by corporate actors
11. Identify the key challenges faced by practitioners and the importance of reimagining training to better prepare them for this work
12. Forge research partnerships with new disciplines to develop more open communication styles in both individual and population level attention to alcohol
13. Stop thinking about alcohol in isolation from other parts of people’s lives
14. Seize opportunities to study brief interventions in the context of major policy changes, including whether such programmes have been involved in their instigation
15. Reframe brief interventions programmes; study rather than assume they are beneficial to population health, and examine whether they have unintended consequences such as widening health disparities

## Data Availability

Not applicable.

## Abbreviation

SBI: Screening and Brief Intervention
