# Correction to: Reimagining brief interventions for alcohol: towards a paradigm fit for the twenty first century: INEBRIA Nick Heather Lecture 2019: This lecture celebrates the work of Nick Heather in leading thinking in respect of both brief interventions and wider alcohol sciences

**DOI:** 10.1186/s13722-021-00270-6

**Published:** 2021-09-30

**Authors:** Jim McCambridge

**Affiliations:** Department of Health Sciences, Seebohm Rowntree Building, University of York, Heslington, York, YO10 5DD UK

## Correction to: Addict Sci Clin Pract (2021) 16:41 10.1186/s13722-021-00250-w

Following publication of the original article [1], the subheading ‘Main text’ was missed in the abstract section of the article, the whole abstract should be as below:

**Abstract**

**Background:** There is no longer support for the idea that brief intervention programmes alone can contribute meaningfully to the improvement of population health relating to alcohol. As a result, calls for major innovations and paradigm shifts grow, notably among research leaders.

**Main text:** This paper briefly examines the history of the development of the evidence-base from the landmark World Health Organisation projects on Screening and Brief Intervention (SBI) in the 1980s onwards. Particular attention is given to weaknesses in the theorisation of social influence and interventions design, and declining effect sizes over time. Although the old SBI paradigm may be exhausted where it has been applied, it has not been replaced by a new paradigm. Alcohol marketing encourages heavy drinking and today may have more powerful effects on thinking about alcohol, and about alcohol problems, than previously. The nature of the societal challenge being faced in an alcogenic environment in which alcohol is widely promoted and weakly regulated underpins consideration of the possibilities for contemporary evidence-informed public health responses. Evidence-informed perspectives in discourses on alcohol problems need to be strengthened in redeveloping rationales for brief interventions. This process needs to move away from sole reliance on a model based on a two-person discussion of alcohol, which is divorced from wider concerns the person may have. Reimagining the nature of brief interventions involves incorporating digital content, emphasising meso-level social processes based on material that people want to share, and seeking synergies with macro-level population and media issues, including alcohol policy measures.

**Conclusions:** Current versions of brief interventions may be simply too weak to contend with the pressures of an alcogenic environment. A new generation of brief interventions could have a key role to play in developing multilevel responses to the problems caused by alcohol.

**Keywords:** Alcohol, Brief interventions, Primary care, Screening, Public health, Alcohol marketing, Alcohol policy, Alcohol industry

The original paper has been updated.
